# Freeze–Thaw Cycle Durability and Mechanism Analysis of Zeolite Powder-Modified Recycled Concrete

**DOI:** 10.3390/ma17112671

**Published:** 2024-06-01

**Authors:** Teng Yu, Yimeng Zhang, Liang Cao, Peng Cao, Changjun Zhou, Shenglong Gu

**Affiliations:** 1 School of Civil Engineering and Water Resources, Qinghai University, Xining 810016, China; yu2017@qhu.edu.cn (T.Y.); 19916363628@163.com (Y.Z.); 2 Laboratory of Ecological Protection and High Quality Development in the Upper Yellow River, Xining 810016, China; 3 Key Laboratory of Water Ecology Remediation and Protection at Headwater Regions of Big Rivers, Ministry of Water Resources, Qinghai University, Xining 810016, China; 4 Faculty of Architecture, Civil and Transportation Engineering, Beijing University of Technology, Beijing 100124, China; liangcao988@126.com; 5 School of Infrastructure Engineering, Dalian University of Technology, Dalian 116024, China; zhouchangjun@dlut.edu.cn

**Keywords:** zeolite powder, recycled concrete, freeze–thaw, mechanical properties, mechanism analysis

## Abstract

The inferior mechanical performance and freeze–thaw (FT) resistance of recycled concrete are mostly due to the significant water absorption and porosity of recycled coarse particles. In this study, different dosages of zeolite powder were used in recycled concrete. A series of macroscopic tests were used to evaluate the workability and FT durability of zeolite powder-modified recycled concrete (ZPRC). X-ray diffraction (XRD) and scanning electron microscopy (SEM) were used to reveal the micro-mechanisms of FT resistance in ZPRC. The results show that the increase in zeolite powder content leads to a decrease in the slump and water absorption of ZPRC. Additionally, ZPRC with 10% zeolite powder has superior mechanical characteristics and tolerance to FT conditions. The higher strength and FT resistance of the ZPRC can be attributed to the particle-filling effect, water storage function, and pozzolanic reaction of zeolite powder, which results in a denser microstructure. The particle-filling effect of zeolite powder promotes the reduction of surface pores in recycled coarse aggregates (RCAs). The water storage function of zeolite powder can provide water for the secondary hydration of cement particles while reducing the free water content in ZPRC. The pozzolanic reaction of zeolite powder can also promote the generation of hydrated calcium silicate and anorthite, thereby making the microstructure of ZPRC more compact. These results provide theoretical guidance for the engineering application of recycled concrete in cold regions.

## 1. Introduction

Recycled concrete, as an environmentally friendly material, can not only effectively reduce the negative impact of construction waste on the environment but also decrease the construction industry’s reliance on natural resources such as natural stone and river sand [1,2,3,4]. Previous studies have shown that the mechanical properties of recycled coarse aggregates (RCAs) are comparable to those of natural coarse aggregates [5]. However, the high water absorption of RCAs and the high porosity of the attached mortar can result in weaker mechanical performance and FT durability of recycled concrete [6,7,8]. Therefore, relevant measures must be urgently adopted to improve the mechanical properties and FT resistance of recycled concrete.

Physical, chemical, and biological treatment measures have been applied to recycled concrete to enhance its mechanical properties and FT durability. The pozzolanic reaction of supplementary cementitious materials can produce calcium–silicate–hydrate (C-S-H) gel, thereby promoting a denser microstructure of recycled concrete [9,10,11]. The high pozzolanic reaction from nano SiO_2_ and particle-filling effect can promote the generation of more C-S-H gel, thereby increasing the stiffness and strength of the interface transition zone (ITZ) between the recycled coarse aggregate and fresh mortar [12,13,14]. Coating recycled aggregates with graphene oxide can promote the generation of more C-S-H gel at the junction of cement between recycled aggregates and fresh mortar under the action of hydrophilic functional groups, thereby reducing the porosity of ITZ and increasing the elastic modulus and stiffness [15,16,17]. The CO_2_ curing treatment can not only effectively fill the pores and internal micro-cracks of RCAs but also increase the hardness of the ITZ [18,19,20,21]. Acid immersion treatment can remove the mortar attached to the surface of RCAs, reducing the porosity of the ITZ between them and the cement matrix [22,23]. Pre-treating RCAs with microorganisms can induce the filling of biologically induced calcium carbonate on the surface of RCAs, making them rougher and enhancing their bonding with mortar [24,25,26]. Based on the above treatment measures, the mechanical performance and FT durability of recycled concrete can be significantly improved. However, these treatment methods usually increase construction costs and processes and, thus, are not widely used in actual projects.

Natural zeolite powder has been used to improve the various properties of concrete due to its characteristics of low carbon environmental protection [27], cation adsorption [28,29], and internal curing [30]. Studies have shown that different types of zeolite powders have a similar effect to ordinary pozzolanic materials in concrete [31]. By reducing the thickness of the ITZ, it effectively improves the microstructure of concrete, thereby enhancing its mechanical properties and durability [32,33]. Furthermore, the porous structure of zeolite powder can confer a water storage function, which can provide water for the secondary hydration of cement particles in the later stage of ITZ, thereby enhancing the FT resistance of concrete in the long term [34,35]. Recently, Bai et al. [36] also used zeolite powder in recycled concrete and found that the pozzolanic reaction of zeolite powder can promote the production of more secondary hydration products in the ITZ, thereby improving the compactness of the microstructure of concrete. For the above analyses, the water storage function of zeolite powder and the pozzolanic reaction were expected to improve the FT resistance performance of recycled concrete. Additionally, the addition of zeolite powder can also reduce the amount of cement in recycled concrete, making the designed recycled concrete more environmentally friendly in terms of carbon emissions.

The above studies have shown that zeolite powder has a good effect on the performance enhancement of ordinary concrete and recycled concrete. However, there is currently limited research analyzing the FT resistance performance of natural zeolite powder-modified recycled concrete (ZPRC). Furthermore, the synergistic mechanism among zeolite powder, cement, and RCAs has not been elucidated. This is extremely detrimental to the widespread application of ZPRC in cold regions.

The objectives of this study were to investigate the influence of natural zeolite powder on the mechanical properties and FT durability of recycled concrete and analyze the synergistic mechanism among zeolite powder, cement, and RCAs. The specific chapter arrangement is as follows. Section 3.1 describes a slump test conducted to evaluate the effects of zeolite powder addition on the workability of recycled concrete. Section 3.2 describes water absorption and FT cycle tests conducted to analyze the FT durability of ZPRC. Section 3.3 describes compressive and flexural strength tests performed to evaluate the mechanical properties of ZPRC with different FT cycle numbers. Section 3.4 describes XRD conducted to analyze the phase composition of ZPRC with different zeolite powder contents. Section 3.5 describes SEM, used to observe the microstructural characteristics of ZPRC and reveal the microstructural mechanism of zeolite powder. This study provides a new solution for enhancing the FT durability of recycled concrete.

## 2. Materials and Methods

### 2.1. Materials

**Figure 1:**
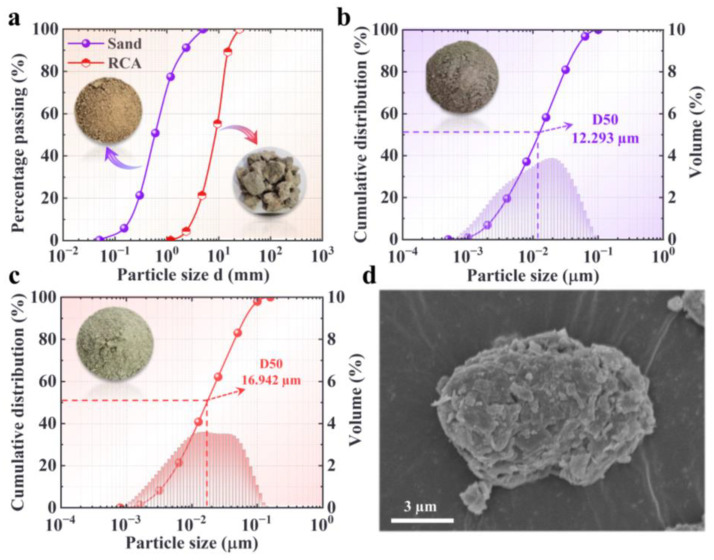
Raw material properties. (**a**) Gradation curves of sand and RCA. (**b**) Gradation curve of cement. (**c**) Gradation curve of zeolite powder. (**d**) Typical microstructure of zeolite particle.

The recycled coarse aggregate consisted of crushed clay bricks and concrete. The size distribution of the recycled coarse aggregate is shown in Figure 1a. The fine aggregate used was machine-made sand with a fineness modulus of 2.6; the size distribution is presented in Figure 1a. Ordinary Portland-type cement, 42.5 MPa, was used, and the size distribution is shown in Figure 1b. Compared to synthetic zeolite [37] and modified zeolite [38], natural zeolite [39] is prepared simply and more cheaply. Therefore, the natural zeolite used in this study was clinoptilolite sourced from a mine in Qinghai Province, China. The size distribution of clinoptilolite is shown in Figure 1c. Furthermore, the microstructure of the zeolite particles was obtained based on SEM (Figure 1d) to provide a reference for the subsequent analysis of the mechanism of action of zeolite powder. The main chemical compositions of the cement and zeolite powder were obtained based on X-ray fluorescence spectroscopy analysis and the results are presented in Table 1. Tap water was used in mixing the ZPRC. Additionally, a polycarboxylic superplasticizer was used in the mixing of the ZPRC to improve its workability.

Zeolite powder was used to replace cement at 0%, 5%, 10%, 15%, 20%, and 25% by mass in this study to investigate the influence of zeolite powder on the mechanical properties and FT durability of recycled concrete. Based on this, this study designed 6 different mix proportions of ZPRC, as presented in Table 2. The sand and water–cement ratios of each mix proportion were fixed at 0.41 and 0.40, respectively.

### 2.2. Sample Preparation

**Figure 2:**
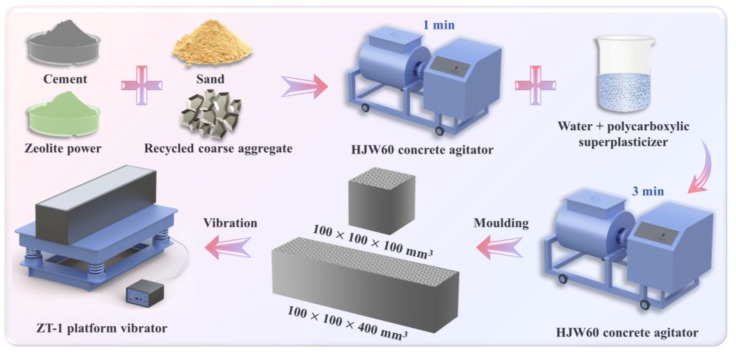
Sample preparation process.

The HJW-60 type concrete mixer (Cangzhou Kexing Instrument and Equipment Co., Ltd., Cangzhou, China) was used for mixing ZPRC. The detailed preparation process of the specimens is shown in Figure 2.

Firstly, cement, zeolite powder, sand, and recycled coarse aggregate were poured into the mixer together and mixed for 1 min. Then, the polycarboxylic superplasticizer solution was poured into the mixer and mixed for an extra 3 min. After mixing, the fresh concrete was poured into 100 mm × 100 mm × 100 mm cubic molds and 100 mm × 100 mm × 400 mm rectangular molds. A vibrating table with the model ZT-1 (Beijing Aerospace Huayu Test Instrument Co., Ltd., Beijing, China) was used for compacting the specimens. Finally, all compacted specimens were placed in a standard curing room for 28 days.

### 2.3. Test Methods

#### 2.3.1. Slump Test

A slump test of fresh ZPRCs with different zeolite powder contents was carried out following the Chinese standard GB/T 50080-2016 [40].

#### 2.3.2. Water Absorption Test

In this study, 100 mm × 100 mm × 400 mm samples without FT cycles were used for water absorption tests, according to the Chinese standard GB/T 50081-2019 [41]. Firstly, the dry mass values of the samples were measured. The distance between samples needed to be no less than 15 mm and the distance above the samples needed to be no less than 20 mm. Secondly, the samples were immersed in tap water for 4 days and weighed daily until a constant mass was achieved. Then, the samples were taken out and wiped to a surface dry condition. Finally, the total mass of the concrete was measured and the value of water absorption was obtained according to Equation (1) [42]:

(1) $$W_{a}=\frac{m_{s}-m_{d}}{m_{d}}\times 100\%$$

where *W*_a_ is the water absorption of the specimen, *m*_s_ is the mass of the specimen when saturated with water, and *m*_d_ is the mass of the specimen when dried.

#### 2.3.3. FT Cycle Test

**Figure 3:**
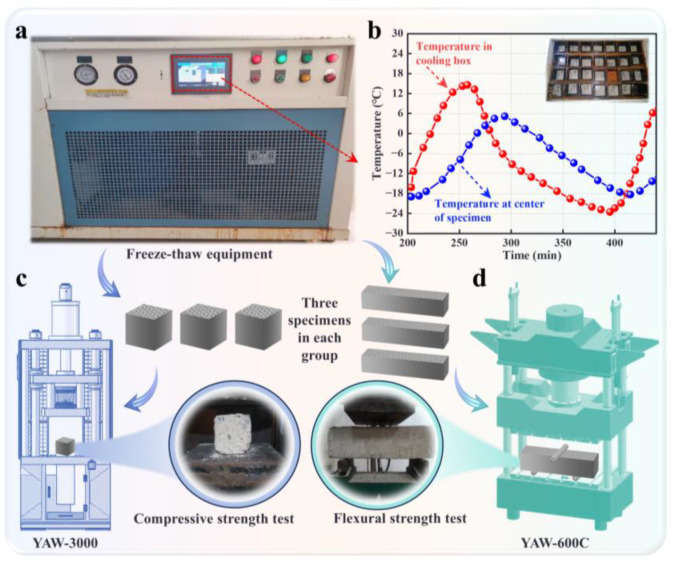
FT and strength tests. (**a**) FT equipment. (**b**) The temperature–time curves of both the cooling box and the specimen center. (**c**) Compressive strength test. (**d**) Flexural strength test.

In this study, samples with dimensions of 100 mm × 100 mm × 100 mm and 100 mm × 100 mm × 400 mm were used. The FT equipment used for the test is shown in Figure 3a. FT cycles were set at 0, 50, 100, 150, and 200. Each FT cycle ran for approximately 4 h. The specific testing procedure followed the “rapid freezing method” outlined in the Chinese standard GB/T50082-2009 [43]. The temperature–the time curves of both the cooling box and the specimen center are illustrated in Figure 3b. The mass loss rate of the specimens after freeze–thaw cycles was calculated according to Equation (2):

(2) $$W_{n}=\frac{m_{0}-m_{n}}{m_{n}}\times 100\%$$

where *W*_n_ is the mass loss rate of the specimen after N freeze–thaw cycles, *m*_0_ is the quality of the specimen before the freeze–thaw cycles, and *m*_n_ is the mass of the specimen after N freeze–thaw cycles.

#### 2.3.4. Compressive and Flexural Strength Tests

Universal testing machines with model YAW-600C (Jinan Docer Testing Machine Technology Co., Ltd., Jinan, China) were used to test the compressive and flexural properties of the ZPRC, according to the Chinese standard GB/T 50081-2019 [41]. The detailed testing procedures are shown in Figure 3c,d. The displacement rate during testing was 5 kN/s. After testing, the average of three measurement results was taken as the representative value of the sample strength.

#### 2.3.5. SEM Analysis

The crushed samples were collected and dried in a 60 °C drying oven [44,45]. After the samples were completely dried, some particles were randomly selected for SEM analysis. The SEM model Nova Nano SEM450 (FEI, Pittsburgh, PA, USA) was used to characterize the microstructure of the samples. Specifically, all samples were gold-coated to enhance conductivity.

#### 2.3.6. XRD Analysis

The dried samples were ground into a powder using a mortar and pestle and sieved through a 45 μm sieve to ensure testing accuracy [46,47]. The XRD analysis was conducted using the X’Pert3 power diffractometer (PANalytical B.V., Almelo, The Netherlands). The XRD analysis used a Cu/Ka radiation source with a step size of 0.01°, scanning speed of 2θ = 1.0°/min, and scanning range of 5–60° [48]. Finally, The International Centre for Diffraction Data was used to analyze the XRD patterns to determine the mineral phase composition of the different specimens.

## 3. Results and Discussion

### 3.1. Slump Test Results

**Figure 4:**
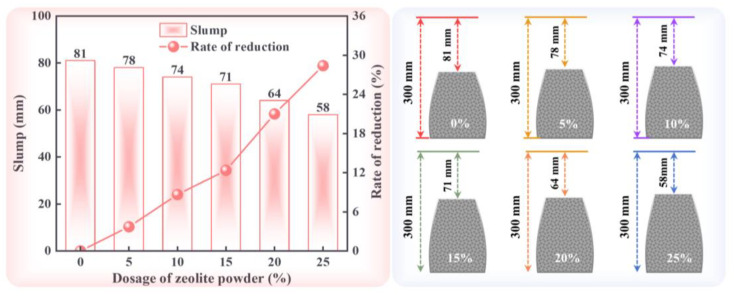
Slump test results and schematic diagrams.

Figure 4 presents the slump values of ZPRCs and the corresponding schematic diagram. It can be clearly observed that the slump of the ZPRC gradually decreased with increasing dosages of zeolite powder. Furthermore, the slump of the ZPRC with a 25% zeolite powder dosage was 58 mm, which was 28.39% lower than that of samples without zeolite powder. This indicates that zeolite powder reduces the workability of recycled concrete. The main reason for this is the high porosity of zeolite powder, which absorbs water in the concrete, and the large surface area of zeolite powder, which increases the inter-particle viscosity [27]. Additionally, in this study, all ZPRCs with different dosages of zeolite powder had the same dosage of water reducer, which resulted in increases in concrete viscosity as the zeolite powder dosage increased. Moreover, a previous study [32] also found similar results, whereby the higher the percentage of cement replaced by zeolite powder, the more a superplasticizer was required to maintain the slump of the concrete. Therefore, when using zeolite powder in recycled concrete, it is recommended to adjust the dosage of the water reducer accordingly to ensure good workability.

### 3.2. Water Absorption and FT Durability

**Figure 5:**
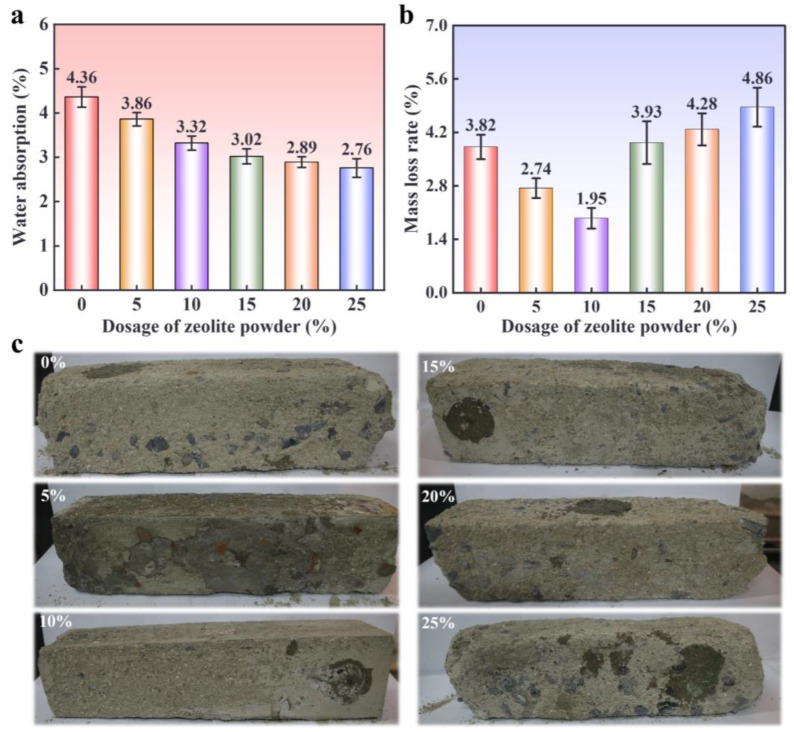
(**a**) Water absorption test results. (**b**) Mass loss rate of specimens with different zeolite powder dosages at 100 FT cycles. (**c**) Surface morphology of specimens with different zeolite powder dosages at 100 FT cycles.

Figure 5a presents the water absorption rate of the ZPRC with different zeolite powder dosages. It was observed that the increase in zeolite powder dosage caused a continuous decrease in the water absorption of the ZPRC. This is similar to the research results of Nagrockiene and Girskas [42], who added zeolite powder to ordinary concrete. With a zeolite powder dosage of 25%, the water absorption rate of the ZPRC was 2.76%, which was 36.70% lower than that of samples without zeolite powder. One of the main reasons for the above phenomenon is the water storage function of zeolite powder, which kept the internal humidity of the sample in a relatively stable state after molding [30,34]. Furthermore, the particle-filling effect of zeolite powder partially occupied the liquid phase space inside the sample. Therefore, the amount of water absorbed by the sample from the external environment was reduced due to the water storage function and particle-filling effect of zeolite powder. This resulted in lower water absorption rates for samples with higher zeolite powder dosages.

Figure 5b shows the mass loss of samples with different zeolite powder dosages after 100 FT cycles. Notably, in this study, the surface morphology of the ZPRC showed no significant damage after 50 FT cycles, while, at 150 and 200 FT cycles, the surfaces of the concrete samples in each group were severely damaged and could not be compared. It can be clearly observed from Figure 5b that for samples without zeolite powder, the mass loss after 100 FT cycles was 3.82%. When the zeolite powder dosage was 5%, the mass loss of the sample was 3.22%. Samples showed the lowest mass loss of 1.95% when the zeolite powder dosage was 10%. However, the increase in zeolite powder dosage caused a decrease in the mass loss of the samples when the dosage of zeolite powder exceeded 10%. One sample exhibited the highest mass loss of 4.86% when the zeolite powder dosage was 25%. The above results indicate that a suitable zeolite powder dosage can significantly enhance the freeze–thaw cycle resistance of recycled concrete. The same conclusions were also obtained by Zheng et al. [34] regarding the improvement of the freeze–thaw durability of ordinary concrete by zeolite powder, but they did not apply the optimum zeolite powder dosage.

From Figure 5c, it can also be noted that the surface morphology changes of the samples were similar to the above-mentioned results for mass loss. For samples with a zeolite powder dosage of 0%, the surface mortar fell off almost completely, exposing the recycled aggregate. When the zeolite powder dosage was 5% and 15%, some of the surface mortar of the sample peeled off, and the overall appearance was relatively intact compared to samples with a 0% zeolite powder dosage. However, with a zeolite powder dosage of 10%, there was almost no mortar detachment on the surface of the sample and the overall appearance remained intact. When the zeolite powder dosage exceeded 15%, the surface appearance and overall damage to the sample became serious. With a zeolite powder dosage of 25%, the surface mortar detachment of the sample was most severe, and there was an overall block loss. Interestingly, the mass loss of the ZPRC was greater with 20% and 25% zeolite powder dosages, but its surface damage was similar to the sample without zeolite powder incorporation. This demonstrates that zeolite powder had a positive effect on maintaining the surface morphology of the specimens after FT cycles.

The above results indicate that the addition of zeolite powder significantly improves the FT resistance of ZPRC. Moreover, this improvement effect is also more noticeable with an increase in the zeolite powder dosage. This is mainly because the water storage function of zeolite powder can promote the secondary hydration of cement and the pozzolanic reaction, producing hydration products that can fill the ITZ between recycled aggregates and mortar [36,49]. Additionally, the particle-filling and water storage functions of zeolite powder also reduce the free water content inside ZPRC, thereby reducing the damage of ice crystal expansion to the microstructure [50,51]. This is also consistent with the results of the ZPRC water absorption test described above. These effects significantly improve the mass loss and surface morphology damage of ZPRC after FT cycles.

### 3.3. Mechanical Properties of ZPRC

**Figure 6:**
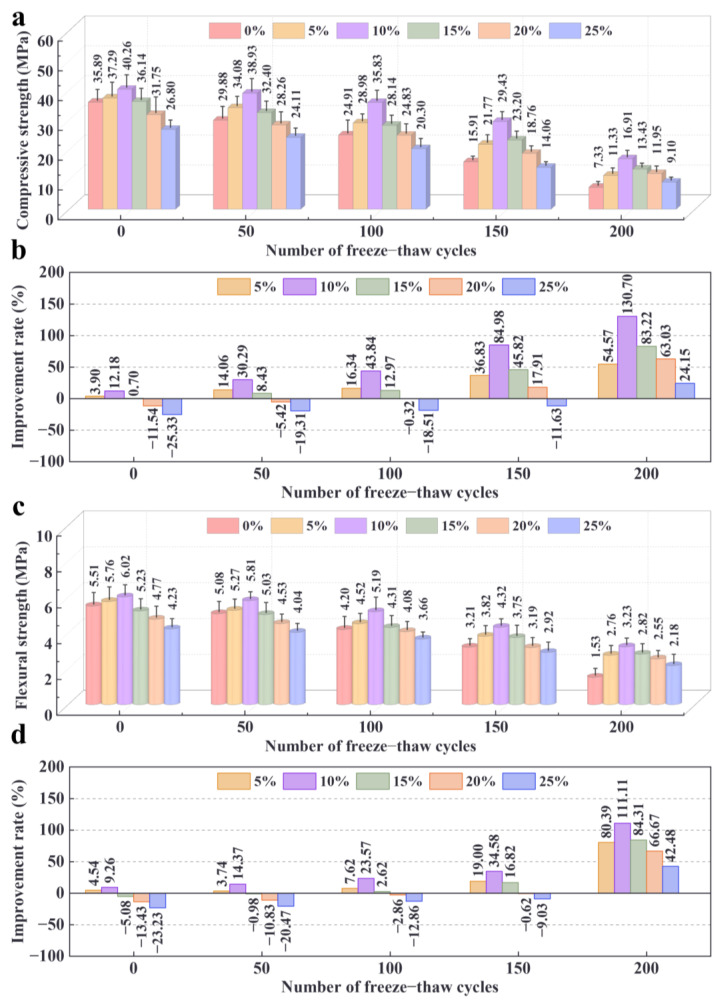
Strength test results. (**a**) Compressive strength. (**b**) Improvement rate of ZPRC compressive strength with different zeolite powder dosages. (**c**) Flexural strength test results. (**d**) Improvement rate of ZPRC flexural strength with different zeolite powder dosages.

Figure 6a presents the results of the compressive strength tests of the samples. Overall, the compressive strength of the samples in each group decreased as the number of FT cycles increased. This was primarily attributed to the pores in the specimen being filled with water and the expansion caused by the freezing of the water destroying the pore structure, leading to a reduction in the strength of the ZPRC [52,53,54]. From Figure 6a, it can also be observed that as the amount of zeolite powder increased, the compressive strength of the specimens at the same number of FT cycles showed a tendency to increase and then decrease. Moreover, the ZPRC had the highest compressive strength with a 10% zeolite powder dosage for all FT cycles. This is similar to the findings of Zolghadri et al. [55], who analyzed the mechanical properties of self-consolidating concrete mixed with zeolite powder. Furthermore, similar conclusions that zeolite powder can reduce the strength loss of recycled concrete after freeze–thaw cycles can be found in the study by Zheng et al. [34] on zeolite powder addition to improve normal concrete. However, the difference is that their study showed that zeolite-cured concrete had poorer frost resistance in the early stages of freeze–thaw cycles and better long-term frost resistance.

Figure 6b presents the improvement effect of zeolite powder on the compressive strength of ZPRC with different numbers of FT cycles. It is remarkable that the loss of strength of the specimens resulting from FT cycles could be significantly improved by the zeolite powder. Moreover, the improvement effect became more pronounced with an increase in the number of FT cycles. It can also be noticed from Figure 6b that a zeolite powder dosage of 10% showed the best improvement in the compressive strength of ZPRC after FT cycles. Zeolite powder could improve the compressive strength of ZPRC up to 130.70% at 200 FT cycles. Figure 6c,d present the flexural strength results and strength enhancement rate of the ZPRC. Similarly, the flexural strength test results of the ZPRC had the same trend as that of compressive strength (Figure 6). However, zeolite powder was slightly less effective in enhancing the flexural strength of ZPRC after FT cycles than the compressive strength.

The improvement mechanism of zeolite powder was mainly due to the water retention function, which facilitated the occurrence of the secondary hydration of cement particles and the pozzolanic reaction in the ZPRC [56]. This resulted in a denser ITZ between the recycled coarse aggregate and the mortar filled with hydration products from the above reactions. Additionally, the free water content inside the ZPRC was also reduced owing to the water storage function of the zeolite powder, which led to a reduction in damage to the microstructure caused by free water icing. However, the reason for the improvement effect of zeolite powder on the mechanical properties of ZPRC with the increasing number of FT cycles is still to be further investigated.

In summary, the strength variation in the above ZPRC corresponded to the mass loss and surface morphology changes of the samples after FT cycles, as described in Section 3.2. Combined with the above test results, it is recommended that about 10% of zeolite powder should be used to replace cement in recycled concrete in practical projects.

### 3.4. The Phase Composition of the ZPRC

**Figure 7:**
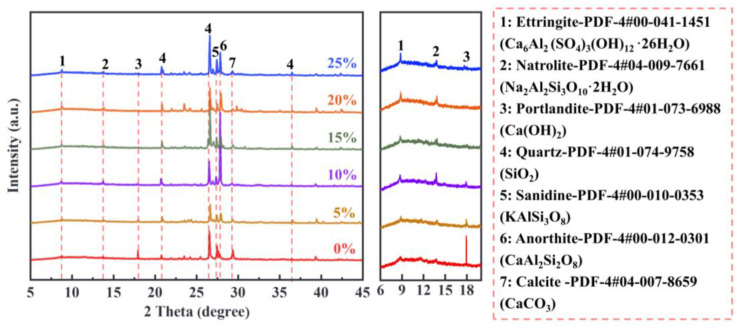
Mineral compositions of specimens with different zeolite powder dosages.

Figure 7 depicts the mineral compositions of the ZPRC with varying zeolite powder contents. It can be observed that with a 0% zeolite powder content, the main hydration products in the samples were ettringite, portlandite, and C-S-H gel. Due to its amorphous nature [57], the characteristic diffraction peaks of the C-S-H gel were not detected. It can also be observed from Figure 7 that the diffraction peak of portlandite was hardly detected in the ZPRC after the dose of the zeolite powder exceeded 10%. Interestingly, the diffraction peak height of ettringite in the ZPRC with a 10–25% zeolite powder dosage was significantly higher than that of the specimens without zeolite powder incorporation. This indicates that zeolite powder has a promoting effect on the generation of ettringite. The reason for this phenomenon is that the pozzolanic reaction products of the aluminum phase in the zeolite powder may react with CaSO_4_ in the recycled aggregate, which promotes the generation of ettringite [58,59]. The specific reaction process is described below [60,61,62,63].

(3) $$3Ca{\left(OH\right)}_{2}+2SiO_{2}\to 3CaO\cdot 2SiO_{2}\cdot 3H_{2}O.$$

(4) $$3Ca{\left(OH\right)}_{2}+Al_{2}O_{3}\to 3CaO\cdot Al_{2}O_{3}\cdot 6H_{2}O$$

(5) $$Ca_{3}Al_{2}O_{6}+\;3\mathrm{Ca}{SO}_{4}\cdot 2H_{2}O+\;26H_{2}O\to Ca_{3}Al_{2}O_{6}\cdot 3\mathrm{Ca}{SO}_{4}\cdot 32H_{2}O$$

The diffraction peaks of natrolite and anorthite could also be detected in the ZPRC owing to the incorporation of zeolite powder, as illustrated in Figure 7. This indicates that the pozzolanic reaction of zeolite powder can also promote the formation of new hydration products in ZPRC. Additionally, it is notable that the natrolite diffraction peak showed no significant change with increasing zeolite powder content. However, the anorthite diffraction peak was significantly enhanced when the zeolite powder content reached 10%. Combining the aforementioned FT cycling and mechanical test results, it can be inferred that the formation of anorthite is the main reason that zeolite powder can improve the mechanical performance and FT cycling resistance of ZPRC.

### 3.5. Microstructural Characteristics of ZPRC

**Figure 8:**
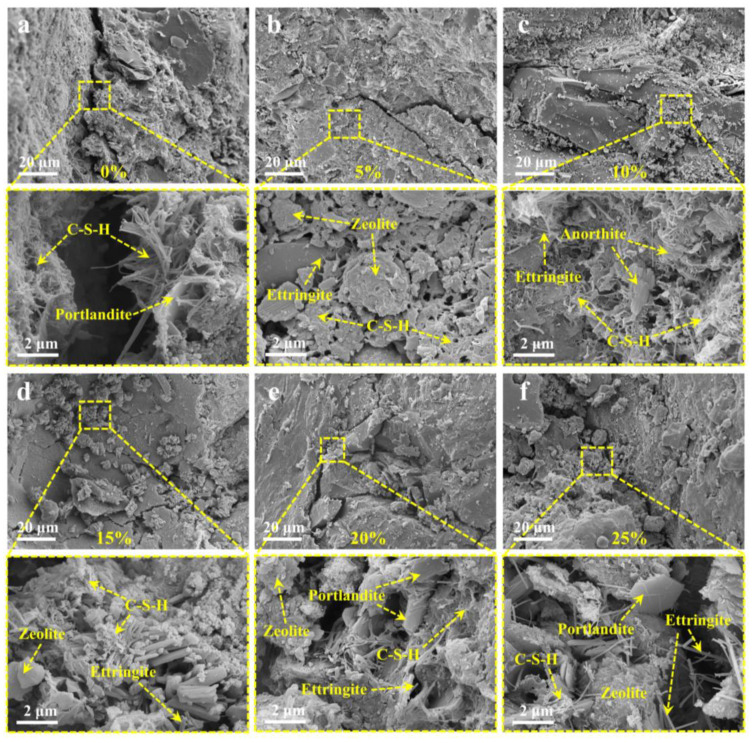
Micro- and meso-structural characteristics of specimens after 100 FT cycles. (**a**) 0%. (**b**) 5%. (**c**) 10%. (**d**) 15%. (**e**) 20%. (**f**) 25%.

Figure 8 presents the microstructural characteristics of the ZPRC with different zeolite contents. It is particularly noted here that the mineral types presented in Figure 8 were determined on the basis of typical hydration product microstructural characteristics, raw material microstructural morphology, and XRD analysis results. From the microstructure of the ZPRC, the crack width between the aggregate and the paste was the largest when the zeolite powder content was 0% (Figure 8a) and 25% (Figure 8f). Next were the specimens with zeolite powder contents of 5% (Figure 8b) and 20% (Figure 8e). The crack width was the smallest for the specimens with zeolite powder contents of 10% (Figure 8c) and 15% (Figure 8d). The above phenomenon is consistent with the results of the FT resistance and mechanical properties of the ZPRC in Section 3.2 and Section 3.3. This also reveals at the microscale that zeolite powder at a content of 10% provides ZPRC with optimal mechanical properties and FT durability mechanisms. That is, the particle filling of zeolite powder and pozzolanic reaction products have a densifying effect on the microstructure of ZPRC. However, this effect is also limited by the zeolite powder content.

From a microscopic perspective, when the zeolite powder content was 0% (Figure 8a), the microstructure of ZPRC mainly consisted of C-S-H gel and Portlandite. Furthermore, the C-S-H gel was unable to bridge the cracks on both sides after FT cycles (Figure 8a). This was a direct cause of the strength reduction of the specimens after experiencing FT cycles [64]. When the zeolite powder content was 5% (Figure 8b), the presence of zeolite powder particles could be observed, and, around these particles, a small amount of ettringite and C-S-H gel could be seen. The C-S-H gel bridged the surrounding minerals in a reticulated form. The ZPRC samples had the most compact microstructure when the zeolite powder content was 10% (Figure 8c). In this microstructure, the presence of reticulated C-S-H gel, ettringite, and anorthite could be noted, and a large amount of reticulated C-S-H gel wrapped and bridged the surrounding structure. Combined with the results of the XRD analysis, the role of anorthite was observed to be spatial filling and bridging. This also provided a microscopic explanation for the higher strength and improved FT durability of the ZPRC specimens with a 10% zeolite content.

Figure 8d–f show that an excessive zeolite powder content led to a reduced reticulated C-S-H gel structure, thereby preventing effective bridging between structures. This resulted in a looser microstructure of ZPRC when the zeolite powder content was 15–25%. This also provided a microscopic explanation for the decrease in the mechanical performance and FT resistance of the specimens when the zeolite powder content exceeded 10%. The main reason for the reduced C-S-H gel structure was primarily due to the substitution of zeolite powder, leading to a decrease in the cement content in the ZPRC, resulting in a reduction of C-S-H gel in the specimens. Additionally, the reduction of cement hydration products also lowered the alkaline environment in the specimens, thereby inhibiting the pozzolanic reaction of the zeolite powder [65], resulting in a further reduction in C-S-H gel. Moreover, Figure 8d–f show the presence of needle-like calcite crystal, similar to the results of XRD. However, due to the relatively small amount of ettringite generated, its filling and bridging effect on the microstructure was limited and therefore could not effectively enhance the mechanical performance and FT durability of the ZPRC.

## 4. Conclusions

This study investigated the influence of zeolite powder on the workability, mechanical properties, and FT durability of recycled concrete. The potential mechanism of zeolite powder in improving the interfacial properties between the recycled coarse aggregate and fresh mortar was also revealed. The following main conclusions were drawn.

1. The addition of zeolite powder increases the viscosity of recycled concrete, leading to a gradual decrease in the slump of ZPRC as the zeolite powder content increases.
2. Zeolite powder reduces the water absorption of ZPRC and improves FT durability and mechanical properties at dosages of 0 to 10%. In this study, the lowest specimen mass loss of 1.95% and the best compressive and flexural strength enhancement of 43.84% and 23.57% after 100 FT cycles were obtained with a zeolite powder dosage of 10%.
3. The addition of zeolite powder promotes the formation of a new hydration product, anorthite, which has a positive effect on the mechanical performance and FT durability of ZPRC.
4. When the zeolite powder content is 10%, the interconnection of C-S-H gel, ettringite, and anorthite in specimens makes the microstructure most dense. However, when the zeolite powder content is 15–25%, the C-S-H gel in ZPRC decreases, causing a loose microstructure.
5. The mechanism of action of zeolite powder, including its particle-filling effect, water storage function, and pozzolanic reaction. The above effects can make the microstructure of ZPRC denser especially when the addition of zeolite powder is 10%.
6. Overall, the results of this study provide a new design scheme for the application of zeolite powder-modified recycled concrete in cold regions. Moreover, due to its low carbon content and cost-effectiveness, zeolite powder can further reduce the carbon emissions and costs of recycled concrete, which is important for low-carbon construction in the construction industry. In future research, we will focus on the dynamic mechanical properties of zeolite powder-modified recycled concrete and its curing effect on radioactive elements in nuclear waste.

## Figures and Tables

**Table 1:** The main chemical compositions of the cement and zeolite powder (wt%).

Type	CaO	SiO_2_	Al_2_O_3_	Fe_2_O_3_	MgO	SO_3_	K_2_O	Na_2_O
Cement	62.25	20.55	5.36	3.95	1.96	2.64	0.72	0.48
Zeolite power	2.62	68.96	12.45	0.92	0.88	0.17	2.68	1.12

**Table 2:** The compositions of 1 m^3^ concrete with different zeolite powder dosages.

Zeolite Ratio (%)	Zeolite Mass (kg)	Cement (kg)	Sand (kg)	Recycled Aggregate (kg)	Water (kg)	Water Reducer (%)
0	0	370	832.5	1189.3	148	1.0
5	18.5	351.5	832.5	1189.3	148	1.0
10	37	333	832.5	1189.3	148	1.0
15	55.5	314.5	832.5	1189.3	148	1.0
20	74	296	832.5	1189.3	148	1.0
25	92.5	277.5	832.5	1189.3	148	1.0

## Data Availability

Data will be made available upon request.
